# The Outcomes of Mental Health Services for Students in Rural Schools

**DOI:** 10.3390/bs16010070

**Published:** 2026-01-04

**Authors:** Jennifer Meek, Janell Walther, HyeonJin Yoon, Mingqi Li, Megan Luther, Jay Jeffries

**Affiliations:** 1 University of Nebraska Public Policy Center, 215 Centennial Mall South, Suite 401, Lincoln, NE 68588-0228, USA; 2 Nebraska Academy for Methodology, Analytics, and Psychometrics (MAP Academy), University of Nebraska-Lincoln, 60 Caroloyn Pop Edwards Hall, Lincoln, NE 68588-0235, USA

**Keywords:** school mental health, internalizing, externalizing, therapeutic services, rural schools, stigma

## Abstract

The location of mental health services in schools increases access for children and youth. This may be especially important in rural communities, where youth have more significant mental health needs and less access to services. Yet, few studies exist that explore the outcomes of student participation in school-based services. The present study evaluates student behavioral health needs and outcomes, as measured by the strengths and difficulties questionnaire (SDQ), of students (*N* = 43) participating in therapeutic mental health services (Tier 3) provided in three rural Midwest communities in the United States. At baseline, SDQ scores indicated that over half of students’ total difficulties scores fell in the Borderline or Abnormal categories, and over 40% of students demonstrated high needs related to emotional problems and hyperactivity. At the conclusion of services, students experienced statistically significant improvements in mean scores (compared to baseline) in total difficulties, externalizing problems, and internalizing problems, and on subscales measuring emotional problems, conduct problems, and hyperactivity. Significant differences were not found in the subscales measuring peer problems and prosocial behavior. High levels of satisfaction with services were also reported. Limitations and conclusions are discussed.

## 1. Introduction

Growing awareness of the need to support youth mental health has been evident in recent years. Even before the COVID-19 pandemic, between 1994 and 2011, the Centers for Disease Control and Prevention reported that up to one in five children and youth in the United States experienced a mental health disorder. Among teenage children, aged 12–17 years, suicide was identified as the leading cause of death (Perou et al., 2013), and rates of youth with major depressive disorder were rising (SAMHSA, Center for Behavioral Health Statistics and Quality, National Survey on Drug Use and Health, 2019). Since the pandemic, more concern for youth mental health has been raised, including a national priority set by the then U.S. Surgeon General (Office of the Surgeon General, 2021) and establishment of a comprehensive mental health action plan by the World Health Organization (World Health Organization (WHO) & United Nations Children’s Fund (UNICEF), 2024). Yet, studies have found that less than half of youth with a mental health disorder receive mental health treatment (e.g., Kase et al., 2017). At such a critical time for development (Smith & Pollak, 2020), youth with unmet needs may face challenges ranging from poorer health outcomes, academic challenges (e.g., Baskin et al., 2010; Rogers et al., 2024), social isolation, and even suicide (Hoover & Mayworm, 2017).

Rural geographies exacerbate the challenges faced by youth with mental health needs. Compared to their urban peers, youth in rural areas have double the rate of suicide and the rate is rising (Hua et al., 2024). Such locations have few mental health providers, making availability of and proximity to mental health care challenging. Those providing care may have limited capacity to provide care or embark on mental health prevention or promotion activities. In rural communities, stigma around mental health, a (perceived or actual) lack of privacy, a distrust of mental health treatment, and insurance challenges are frequent barriers to care. These barriers to mental healthcare in rural areas mean that while children and youth are not only unable to access treatment but, for those that do access treatment, the attrition rate is particularly high in community-based services (Hoover & Mayworm, 2017; Lee et al., 2009).

For these reasons, the provision of mental health services in school settings, which serve students in kindergarten through grade 12 (K-12, i.e., primary and secondary school), is beneficial. This is especially true in rural areas, highlighting the opportunity to improve student well-being but also increase school attendance, increase academic outcomes, promote social connection, and strengthen overall student health. School-based mental health services help with early identification of mental health needs, delivery of mental health services, and integrate treatment with other school-based interventions (Hoover & Bostic, 2021). Additionally, schools are well-suited to be a place for mental health services: students spend a significant portion of their lives at school, and schools can be a source of trust for families. In rural areas, schools are situated to address the lengthy list of barriers to mental healthcare students experience, as schools are the place where families already receive many of their services and supports (Ash et al., 2025; Siceloff et al., 2017). Likewise, there are benefits for the students including improved treatment participation, decreased stigma around mental health, and positive academic and social-emotional functioning (Hoover & Bostic, 2021; Kase et al., 2017).

There are many approaches to integrating mental health services into school settings. The Comprehensive School Mental Health Systems (CSMHS) model moves beyond service provision, and expands by implementing tiered mental health promotion, intervention, and services; creating cross-disciplinary collaborative teams to address school mental health needs and continuous monitoring of data and outcomes to assess quality, progress, and sustainability (Hoover & Bostic, 2021). This systemization of school-based mental health provides student mental health services ranging from universal mental health promotion (e.g., Tier 1—social-emotional curriculum, school-wide mental health screening), to early interventions for selected students at risk (e.g., Tier 2—small-group social skills instruction, brief 1:1 check-ins with identified staff), to intensive intervention for those students with more several needs (e.g., Tier 3—school-based 1:1 therapeutic services). In addition to services, CSMHS also places a focus on creating stability for this system by instituting school mental health teams, formalizing policies and quality monitoring, and identifying funding solutions for long-term sustainability (Hoover & Bostic, 2021). In addition to implementing the CSMHS system, rural schools also add focus on the school–community connection, often leaning on both relationships and informal support to meet the needs of students and families in rural areas (Anderson-Butcher et al., 2017; Collier et al., 2025).

### 1.1. Behavioral Health Outcomes of School-Based Services

School-based interventions, which range in intensity from large group prevention efforts to clinical therapy, have demonstrated positive behavioral health outcomes for students (Kase et al., 2017). Research studies have demonstrated decreases in mental health needs, including symptoms of PTSD and depression (Kase et al., 2017), as well as emotional problems and total difficulties as rated by teachers and parents on the strengths and difficulties questionnaire (Ballard et al., 2014) as a result of school-based mental health interventions. School-based services have also been found to demonstrate small-to-medium effects on child mental health when delivered by school personnel rather than researchers (Sanchez et al., 2018). Social-emotional learning curricula have demonstrated increases in student emotional understanding and prosocial behavior (Gibson et al., 2015). Clinical interventions, including provision of therapeutic services, have improved overall symptoms and functioning (Walter et al., 2019), including internalizing and externalizing behavior (Kase et al., 2017).

#### Student Outcomes in Rural Settings

Few studies have explored the behavioral health outcomes of school-based mental health systems and services for students in rural schools. In one example of such a study, Watabe et al. (2013) examined a mental health program for elementary youth with or at-risk of ADHD in a rural school district. Their study found medium to large effects of parent ratings of students’ behavioral outcomes and smaller effect sizes were found in teacher reports. While not all changes in student behavior were significant, the authors assessed improvement (Watabe et al., 2013).

Likewise, Albright et al. (2013) conducted studies exploring the effects of therapeutic treatment provided to students at the Assessment, Support, and Counseling (ASC) Center located in a rural high school in North Carolina. They found that while 55% of students presenting for treatment did not have clinically significant mental health needs (as measured by the BASC-2), 78% of students exhibited less psychological distress at the conclusion of treatment (Albright et al., 2013). For those students with clinically significant baseline scores, 63% were considered improved or recovered at the end of treatment (Albright et al., 2013). For these same students, baseline scores on the YOQ-30 indicated that 66% of the sample had clinically significant symptoms, and at the final assessment 45% were considered recovered (Michael et al., 2013).

Most recently, Collier et al. (2025) evaluated the impact of an elementary school mental health initiative, called the Pee Dee Resiliency Project, based in South Carolina. Children who received treatment from a school-based clinician experienced a significant decrease in scores, as reported by parents on the Pediatric Symptoms Checklist (PSC; Collier et al., 2025). At the end of services, 77% of parents also reported noticing an improvement in student behavior at home. Students also reported a significant increase in their resilience and both parents and students reported high levels of satisfaction with services (Collier et al., 2025).

These studies provide important contributions to the literature by documenting the outcomes of school-based mental health services, including therapeutic services, in rural settings. Within this context, there are unique challenges to mental health service provision (i.e., provider shortages, distance and travel, stigma), which may make school-based services the only option available to students. However, the two studies that explore the outcomes of therapeutic services are based in North and South Carolina and explore outcomes at one school level (elementary or high school). The purpose of this study is to build on these existing studies to further explore the mental health outcomes of therapeutic services offered as part of a comprehensive school mental health systems, to students in grades K-12 in three rural communities in a Midwestern State (USA). Specifically, this study seeks to answer the following questions:

1. What behavioral health needs are demonstrated by students referred to mental health services, as measured by the strengths and difficulties questionnaire?
2. How do the behavioral health outcomes of students receiving school-based therapeutic services change, from the start (baseline) to end (discharge) of services, as measured by the strengths and difficulties questionnaire?

## 2. Materials and Methods

This study was part of a comprehensive program evaluation examining the implementation and impact of Comprehensive School Mental Health Systems in three rural Midwestern communities in the United States. The State Department of Education (SDE) was awarded a federal grant to provide funding and technical assistance to three rural school districts implementing school mental health programs. The SDE contracted with a University research center to conduct an external evaluation, the purpose of which was to assess the outcomes of grant activities. The evaluation included both process and outcome measures related to educator training, delivery of student prevention and intervention services, implementation of universal screening, and policy development. The evaluation was determined not to constitute Human Subject’s Research by the University’s Institutional Review Board.

### 2.1. Data Collection

The student outcome measures (SOMs) survey tool was developed to measure outcomes for students receiving therapeutic services at a school building (*n* = 13) in one of the three school districts participating in the project. Student mental health needs were identified via universal screeners, teacher or counselor observation, self-referral, or parent referral. Identified student mental health needs were then reviewed by a building-based school mental health teams (e.g., school counselors, psychologists, social workers, therapists, administrators), who determined student referrals to school-based therapeutic services.

Data was collected by mental health services providers at each school site. Students age 11 and older were invited to complete the self-report survey, while caregivers or teachers were invited to provide reports for students age 10 years or younger. When providing consent/assent for mental health services, parents and students were asked if their survey responses could be de-identified and shared with the evaluation team. For those that agreed, providers assigned a unique project ID and submitted the de-identified survey to the evaluation team via a secure online system.

### 2.2. Measures

The SOMs tool used to measure outcomes for these students consists of three components: 25-item scale and 10-question supplement strengths and difficulties questionnaire (SDQ; Goodman, 1997), 6-item social connectedness Scale (SAMHSA’s Performance Accountability and Reporting System (SPARS), 2022), and 4-item service satisfaction scale from the Peabody Treatment Progress Battery (Bickman et al., 2010). These measures were selected as validated scales applied in both clinical and research for youth ranging from early childhood through secondary school. Data was collected at baseline, reassessment (6 months from baseline) and discharge (when a student is no longer receiving services). While the SDQ and social connectedness scale were collected at all points in time, the Peabody Treatment Progress Battery was only collected at reassessment and discharge because items were focused on service satisfaction.

This study evaluates student behavioral health outcomes with the SDQ portion of the SOMs tool. The SDQ assesses subscales related to emotional problems, conduct problems, hyperactivity, peer problems and prosocial behavior. The total difficulties score aggregates all subscales except prosocial behavior, a positively scaled measure. impact scores represented the distress caused by the difficulties experienced, as reported by participants. Externalizing problems (measured via emotional and peer problem items) and internalizing problems (measured via conduct problems and hyperactivity symptom items) were also scored (Table 1). Subscale scores, along with scores for total difficulties and impact, were classified into risk categories. Given the prosocial subscale was a measure of positive behavior, low scores were considered at risk. Categories include close to average, slightly raised (slightly lowered for the prosocial subscale), high (low for the prosocial subscale), and very high (very low for the prosocial subscale). Internalizing and externalizing problems scales are not categorized.

Scale data reliability was assessed using McDonald’s omega (ω) measure of internal consistency for each subscale and composite scale. The McDonald’s ω estimate (McDonald, 1999) does not assume tau-equivalence and is appropriate for unit-weighted scales whose psychometric structure may be congeneric (McNeish, 2018). Estimates were calculated using baseline data from all reporter types and a threshold of ω ≥ 0.70 conveyed adequate reliability. The range, internal consistency, and interpretation for each measure is shown in Table 1. These reliability estimates led to the following decisions: the (a) conduct problems scale (ω = 0.66) was retained because it demonstrated marginally adequate reliability, (b) peer problems scale (ω = 0.56) was not examined due to inadequate reliability; and (c) the total difficulties (ω = 0.72) and internalizing problems (ω = 0.77) composite scales were not examined because they are calculated using peer problems scale items.

### 2.3. Analytic Procedure

SDQ data was analyzed with descriptive statistics to understand student mental health needs at baseline (RQ1). This included analysis of baseline records for 114 students. Descriptive statistics involved measures of central tendency and proportions of each score category (i.e., close to average, slightly raised (slightly lowered for the prosocial subscale), high (Low for the prosocial subscale), and very high (very low for the prosocial subscale)) for SDQ subscales (i.e., emotional problems, conduct problems, hyperactivity, prosocial behavior).

A series of two-level linear mixed-effects models (i.e., multilevel models) were implemented to examine the change in SDQ subscale scores and externalizing symptoms between baseline and discharge (RQ2). The use of multilevel modeling was appropriate to account for the nesting of repeated measurements within participants (Snijders & Bosker, 2011). A total of 43 participants, who completed services and were discharged during the study, were included in the mixed-effects model analyses, with no missing data on the outcome variables for any of these participants. There were 71 students still receiving services, with no discharge record, who were not included in the analyses.

At Level 1, the SDQ subscale scores were predicted as a linear function of time (Baseline = 0, Discharge = 1). At Level 2, participant-level covariates (gender, race, and ethnicity) were entered, where Female, White, and Hispanic or Latinx served as reference groups. The model was fitted using the lme4 package (Bates et al., 2015) in R (R Core Team, 2025). To control for inflation of Type I error due to multiple testing, *p*-values were adjusted using the Benjamini–Hochberg false discovery rate (FDR) procedure (Benjamini & Hochberg, 1995). Marginal, representing the proportion of variance explained by the fixed effects, was calculated using the Nakagawa method (Nakagawa & Schielzeth, 2013). In addition, semi-partial *R*^2^ (*sr*^2^) for the time predictor was also calculated using the PartR2 package (Stoffel et al., n.d.) in R to estimate unique variance in the outcome attributable to time, serving as an effect size measure. The model is specified as in Equation (1):

*Y_ti_ = γ*_00_ + *γ*_10_ *_Time_ + γ*_20 Male_ + *γ*_30 African American_ + *γ*_40 American Indian_ + *γ*_50 Mixed Race_
 + *γ*_60 Non-Hispanic_ + *u*_0*i*_
*+ r_ti_* (1)

where *Y_ti_* is the SDQ domain scores for time *t* for participant *i*; the intercept, *γ*_00_, denotes the mean SDQ domain score at baseline; *γ*_10_ is the slope relating time (Baseline = 0, Discharge = 1) to the SDQ domain score; *γ*_20_ is the mean difference in SDQ domain score between male and female participants at baseline; *γ*_30_, *γ*_40_, and *γ*_50_ represent the mean difference at baseline between African American, American Indian, and Mixed Race participants, respectively, relative to White participants; *γ*_60_ is the baseline mean difference between Non-Hispanic and Hispanic participants; *r_ti_* is residual at time *t* for individual *i*. At the individual level, the intercept was modeled as a random effect (*u*_0*i*_), allowing baseline scores to vary across participants.

### 2.4. Population

This study includes three school districts located in a Midwestern state. All three school districts are located in areas designated as having a shortage of mental health and psychiatry services (Rural Health Advisory Commission, 2019) but are unique in terms of demographics and geography. School District 1 is located in a community of 10,032 people in the west central part of the state, along an interstate corridor. School District 2 is in a town of 7289 people in the southeastern portion of the state, about a one-hour drive from the state’s largest metropolitan area. School District 3 serves a remote ranching community of 2737 people in the north central part of the state. While all three sites preside in rural communities, student enrollments vary from 3299 (School District 1) to 611 (School District 3; see Table 1). School District 1 is also unique from the other two district in terms of student population, with most students identifying as Hispanic/Latino, receiving free or reduced-price lunch, and 41% of students identified as English Language Learners (Table 2).

## 3. Results

### 3.1. Student Behavioral Health Needs

There were 114 SDQ records (82 self-reports, 27 caregiver reports, and 5 teacher reports) completed for students who received individual mental health services from October 2022 to May 2025. At baseline, over 50% of the records fell into the close to average category for conduct problems (62.3%), prosocial (71.1%), and hyperactivity (54.4%). About one-fourth of records fell into very high for emotional problems (26.3%). Most respondents reported very high (52.7%) impact scores, suggesting serious burden from the problems they experienced (Table 3).

Baseline scores, on average, were near the midpoint for emotional problems and hyperactivity. On average, prosocial scores were closer to the scale maximum, and scores related to conduct problems were closer to the scale minimum—both positive outcomes. Externalizing problems scores fell just below the midpoint of the score range (Table 4).

### 3.2. Bivariate Correlations

Correlation analyses indicated that, at baseline, both emotional problems and conduct problems were positively correlated with hyperactivity and externalizing problems (rs ≥ 0.39, *p*s < 0.05). Hyperactivity was also strongly correlated with externalizing problems (r = 0.88, *p* < 0.05). Across time, all outcomes showed significant positive longitudinal correlations with one another from baseline to discharge (rs ≥ 0.47, *p*s < 0.05; Table 5).

### 3.3. Changes in Behavioral Health Outcomes

#### 3.3.1. Emotional Problems

**Figure 1:**
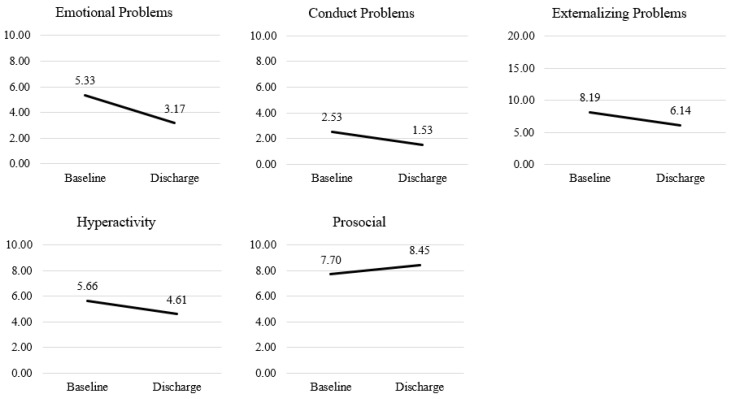
Change in Mean strengths and difficulties questionnaire (SDQ) scores (baseline to discharge).

Participants exhibited a statistically significant decline in emotional problems scores from baseline to discharge (γ_10_ = −2.16, *SE* = 0.33, *sr*^2^ = 0.18; Figure 1). At baseline, male participants had significantly lower emotional problems scores than female participants (γ_20_ = −2.06, *SE* = 0.57; Table 6).

#### 3.3.2. Conduct Problems

Results showed a statistically significant decrease in conduct problems scores from baseline to discharge (γ_10_= −1.00, *SE* = 0.25, *sr*^2^= 0.08). No significant difference in demographic covariates at baseline was found.

#### 3.3.3. Hyperactivity

Participants’ hyperactivity scores significantly decreased from baseline to discharge (γ_10_ = −1.05, *SE* = 0.28, *sr*^2^= 0.05), indicating reductions in hyperactivity behaviors over time. No significant baseline differences were observed across demographic covariates.

#### 3.3.4. Prosocial Behavior

Participants showed a statistically significant increase in prosocial behavior scores from baseline to discharge (γ_10_ = 0.74, *SE* = 0.32, *sr*^2^= 0.04), suggesting that prosocial behaviors improved over the course of the program. At baseline, male participants scored significantly lower on prosocial behavior than female participants (γ_20_ = −1.27, *SE* = 0.48), while Mixed Race participants scored significantly higher than White participants (***Y***_50_ = 1.70, *SE* = 0.67).

#### 3.3.5. Externalizing Symptoms

Participants’ externalizing symptoms scores decreased significantly from baseline to discharge (γ_10_ = −2.05, *SE* = 0.44, *sr*^2^= 0.08), indicating reductions in externalizing behaviors (e.g., aggression) over the course of the program. No significant differences were found across demographic covariates at baseline.

## 4. Discussion

This study first explored the needs of students upon referral to school-based mental health services. Baseline SDQ subscale scores demonstrate that between 48.2% (emotional problems subscale) and 71.7% (prosocial subscale) of students receiving therapeutic services scored in the close to average category. The CSMHS model includes provision of three tiers of mental health supports—mental health prevention and promotion services available to all students (Tier 1), small group intervention (Tier 2), and individual intervention (Tier 3). While the model indicates that Tier 3 services are provided to students with the highest level of need, results suggest otherwise. At baseline, all students did not demonstrate high levels of need, as measured by the SDQ, which aligns with baseline measures in other studies (Albright et al., 2013; Michael et al., 2013). While such findings are contrary to the model, it is unknown how student mental health needs were supported by Tier 1 and Tier 2 interventions prior to the provision of therapeutic services within this data. Future research is needed to understand how students’ needs are met with Tier 1, 2, and 3 services, and whether students participate in other services prior to referral to Tier 3. This research may be especially important in rural contexts, where school staff are over-extended and may not have capacity to provide additional support (Meek Farley et al., 2025). Further exploration into identification and referral practices will also benefit the field. This includes understanding whether and how student behavioral health and academic outcomes may be observed and assessed prior to identification and referral. Additionally, considerations should be made regarding how these practices may differ in rural school districts, where there are limited options for identifying needs or making referrals outside of the school itself, due to a limited number of community providers.

Comparison of baseline and discharge SDQ scores indicate students receiving school-based, therapeutic mental health services in rural school districts significantly improved mental health outcomes. Between baseline and discharge, students demonstrated progress on all SDQ subscales (emotional problems, conduct disorders, hyperactivity, prosocial) and the measure externalizing problems. These findings align with prior research on behavioral health outcomes of school-based services (Ballard et al., 2014; Kase et al., 2017; Sanchez et al., 2018; Walter et al., 2019) and add to known outcomes of therapeutic school-based services in rural locations (Albright et al., 2013; Michael et al., 2013; Collier et al., 2025). Results suggest that the findings of those studies may not be limited by geographic location, student grade level, or specific models of school mental health.

Inherent to these findings is that implementation of CSMHS provides rural youth with access to mental health services (Hoover & Bostic, 2021). The three rural communities participating in the project were selected by State Department of Education based on student mental health needs and limited resources. During the reporting period, project sites established therapeutic services within CSMHS and provided services to over 100 children and youth. It is unknown how these students’ mental health needs would have been met without the project. In this way, it is important to recognize that the project first provided access to mental health services, then, for students who accessed services, positive behavioral health outcomes resulted.

Significant differences in outcomes were found between gender and racial groups on the emotional problems and prosocial subscales. This is a unique finding of this study, as prior research in rural settings did not explore differences between demographic groups. Gender differences are not entirely surprising, as research shows that anxiety and depression (Anderson et al., 2024) and prosocial behavior (Van der Graaff et al., 2018) is more prevalent among female adolescents. While school-based mental health services have the potential to increase student access (Hoover & Bostic, 2021), school-based interventions have also been found to be less effective for underserved populations (Farahmand et al., 2011). Given this, equity must not only be central to school mental health but actively evaluated (Garbacz et al., 2024). Future research must continue to consider diverse student populations receiving services and may use outcomes to identify or develop targeted interventions. It may also be important to consider expanding the demographic variables explored to include measures of socioeconomic status and language, two factors that may limit access to mental health care outside of school. Ultimately, continued exploration of student demographic factors may help to ensure all students benefit from school-based services.

While results demonstrate improvement in student mental health outcomes, it is unknown how these changes relate to academic success. While the existing literature calls for further research to understand both how improved mental health and academic achievement are related (Baskin et al., 2010) and when academic gains may be expected (Michael et al., 2013), a broader understanding of academic needs at baseline may be helpful. It is unknown how the mental health and academic needs of students may be considered in identification and referral processes within school-based mental health. Inclusion of academic needs in these processes may help to explain the number of students who do not meet clinical or risk thresholds for behavioral health needs at baseline, in this and other studies (e.g., Albright et al., 2013, Michael et al., 2013).

Finally, it is important to consider that this and other studies of therapeutic school-based services in rural settings have been conducted as part of larger program evaluations of school-based mental health. The purpose of program evaluation and research are different, with program evaluation informing decision making and supporting learning rather than generating knowledge and theory (Russ-Eft & Preskill, 2009). Given this, these evaluative studies are less likely to meet the literature’s call for rigorous research designs (e.g., experimental designs, multi-level analysis; Ballard et al., 2014) and exploration of complex relationships (e.g., mediators, moderators) between mental health, academic achievement and perceptions of school culture and climate (Michael et al., 2013). Program evaluations are often a requirement of grant-funded projects, as is the case in this study, and while grant funding can help to establish school-based mental health programs, sustainability is a challenge once funding ends (Hoover, 2024). This challenge is especially concerning in rural communities, where schools may be the only option for mental health services (Hoover & Bostic, 2021). It is essential that sustainable sources of funding be made available not only for the provision of school-based mental health in rural locations but also for research to better understand these systems and outcomes in these communities to inform future SBMH efforts.

### Limitations

This study may be limited by its design. As part of a larger program evaluation study, schools and students participating in the project were a part of the grant program and were not selected at random. Students receiving services were identified and referred through their school district’s identified referral pathways, and were not randomly assigned, nor was a control or comparison group identified. While ethical considerations must be made to the use of experimental designs in provision of mental health services (Michael et al., 2013), the study cannot draw causal conclusions related to student improvement.

The study may also be limited by the tools used to collect data on student behavioral health outcomes. While the SDQ includes student, parent, and teacher survey reports, the capacity of service providers and scope of the evaluation only allowed for one report to be collected per student. Therefore, it is unknown how parent and teacher perceptions of student behavior would compare to student reports. Additionally, one subscale of the SDQ (peer problems) conveyed reliability estimates lower than conventional thresholds and was excluded from analysis. Research has documented challenges with the reliability of this scale (Kankaanpää et al., 2023). Yet, exclusion of this scale impacted our ability to analyze results related to total difficulties and internalizing problems, both of which include the peer problems subscale.

## 5. Conclusions

Overall, this study furthers the research on mental health outcomes of school-based mental health services in rural schools. Findings reveal that a small proportion of students referred to school-based mental health services demonstrate high levels of need as measured by the SDQ. However, comparison of baseline and discharge SDQ demonstrates significant improvement across all SDQ subscales. Given the few studies that exist, and the unique challenges related to mental health service provision in rural communities, there is a need for continued research regarding the implementation and outcomes of school mental health. Such studies should expand their focus to explore results by student demographic groups and account for identification and referral processes and practices. Finally, further exploration is needed into the ways in which positive changes in mental health outcomes are related to academic achievement.

## Figures and Tables

**Table 1:** Scoring and interpretation for SDQ measures used.

SDQ Subscales	Possible Range	ω	Interpretation
Emotional problems	0–10	0.84	Higher scores indicate more problems
Conduct problems	0–10	0.66	Higher scores indicate more problems
Hyperactivity	0–10	0.77	Higher scores indicate more hyperactivity
Peer problems	0–10	0.56	Higher scores indicate more problems
Prosocial *	0–10	0.84	Higher scores indicate more prosociality
Total difficulties	0–40	0.72	Higher scores indicate more difficulty
Externalizing problems	0–20	0.73	Higher scores indicate more problems
Internalizing problems	0–20	0.77	Higher scores indicate more problems
Impact	0–10	0.80	Higher scores indicate more interference

Note. For all subscales except for those marked with an *, higher numbers indicate more problems or difficulties; ω = McDonald’s omega internal consistency.

**Table 2:** School district student characteristics, 2024.

PK-12th Enrollment	School District 1 (*N* = 3229)		School District 2 (*N* = 1402)		School District 3 (*N* = 611)	
	*n*	%	*n*	%	*n*	%
Race/Ethnicity						
Asian	18	0.6	4	0.6	4	0.7
Hispanic	2474	76.6	327	23.3	32	5.2
White	400	12.4	1020	72.8	468	76.6
American Indian/Alaska Native	12	0.4	12	0.1	60	9.8
Black or African American	293	9.1	8	0.6	1	0.2
Native Hawaiian or Other Pacific Islander	7	0.2	0	0.0	0	0.0
Two or More Races	25	0.8	41	2.9	46	7.5
Gender						
Female	1507	46.7	660	47.1	302	49.4
Male	1722	53.3	742	52.9	309	50.6
Free/Reduced Lunch	2438	75.5	772	55.1	286	46.8
English Language Learners	1327	41.1	102	7.3	*	*
2024 Graduation Rate		96.8		85.0		94.6

Notes. * Some information is redacted to protect confidential information as required by federal law.

**Table 3:** SDQ results by category at baseline.

SDQ Indicator	Close to Average		Slightly Raised/Lowered		High/Low		Very High/Low	
	N	%	N	%	N	%	N	%
Emotional problems	55	48.2	15	13.2	14	12.3	30	26.3
Conduct problems	71	62.3	21	18.4	14	12.3	8	7.0
Hyperactivity	62	54.4	24	21.1	16	14.0	12	10.5
Prosocial *	81	71.1	16	14.0	10	8.8	7	6.1

Note. * indicates an inverted scale, where ‘high’ scores are ‘low’.

**Table 4:** Mean SDQ scores at baseline.

SDQ Score	*n*	Min	Max	Mean	*SD*
Emotional problems	114	0	10	4.64	2.50
Conduct problems	114	0	10	2.64	1.93
Hyperactivity	114	1	10	5.32	2.09
Prosocial *	114	1	10	7.86	2.00
Externalizing problems	114	2	20	7.95	3.52

Note. For all indicators except for those marked with an *, higher numbers indicate more problems or difficulties.

**Table 5:** Bivariate correlations among the SDQ domain scores for both timepoints.

	1	2	3	4	5
1. T1 Emotional Problems	–				
2. T1 Conduct Problems	0.20	–			
3. T1 Hyperactivity	0.52 ***	0.39 *	–		
4. T1 Prosocial	0.17	−0.13	−0.07	–	
5. T1 Externalizing Problems	0.45 **	0.79 ***	0.88 ***	−0.12	–
T2 Emotional Problems	0.51 ***				
T2 Conduct Problems		0.54 ***			
T2 Hyperactivity			0.66 ***		
T2 Prosocial				0.47 ***	
T2 Externalizing Problems					0.60 ***

Note: *N* = 43. T1 = Baseline. T2 = Discharge. * *p* < 0.05. ** *p* ≤ 0.01. *** *p* ≤ 0.001.

**Table 6:** Mixed-effect model results for changes in strengths and difficulties questionnaire (SDQ) scores (baseline to discharge).

Predictors	Emotional Problems	Conduct Problems	Hyperactivity	Prosocial	Externalizing Problems
	Estimate (*SE*)	Estimate (*SE*)	Estimate (*SE*)	Estimate (*SE*)	Estimate (*SE*)
Intercept, *γ*_00_	5.81 (0.45) ***	2.76 (0.40) ***	5.32 (0.51) ***	7.86 (0.39) ***	8.08 (0.76) ***
Time (Discharge), *γ*_10_	−2.16 (0.33) ***	−1.00 (0.25) ***	−1.05 (0.28) **	0.74 (0.32) *	−2.05 (0.44) ***
*sr*^2^ for Time [95% CI]	0.18 [0.01, 0.36]	0.08 [.04, 0.33]	0.05 [0.00, 0.29]	0.04 [0.00, 0.27]	0.08 [0.00, 0.37]
Demographic Covariates					
Gender (Male), *γ*_20_	−2.06 (0.57) **	−0.20 (0.51)	−1.03 (0.67)	−1.27 (0.48) *	−1.23 (0.98)
Race (African American), *γ*_30_	1.90 (1.26)	0.95 (1.31)	3.00 (1.46)	−1.41 (1.06)	3.96 (2.16)
Race (American Indian), *γ*_40_	1.17 (0.92)	0.09 (0.83)	0.97 (1.07)	−0.09 (0.77)	1.06 (1.58)
Race (Mixed Race), *γ*_50_	−0.36 (0.80)	−0.71 (0.72)	0.64 (0.93)	1.70 (0.67) *	−0.07 (1.37)
Ethnicity (Non-Hispanic), *γ*_60_	−0.26 (0.70)	−0.43 (0.63)	−0.60 (0.82)	0.86 (0.59)	−1.03 (1.21)
Marginal *R*^2^	0.41	0.12	0.20	0.22	0.20

Notes. *N* = 43. For gender, Female is the reference group. For race, White is the reference group. For ethnicity, Hispanic is the reference group. *sr*^2^ = semi-partial *R*^2^. CI = confidence interval. Values of approximately 0.01, 0.09, and 0.25 may be interpreted as small, medium, and large effect sizes, respectively (Cohen, 1988). * *p* < 0.05. ** *p* < 0.01. *** *p* < 0.001.

## Data Availability

The datasets presented in this article are not readily available because of the nature of our evaluation contract with the Nebraska Department of Education. Requests to access the datasets should be directed to jenniferfarley@nebraska.edu.
